# Editorial: Improving crop health: Understanding the interaction mechanisms between crops and their pathogens

**DOI:** 10.3389/fpls.2023.1161154

**Published:** 2023-02-21

**Authors:** Yuheng Yang, Qiong Zhang, Yang Yu, Guotian Li, Shunyuan Xiao, Zhanhong Ma

**Affiliations:** ^1^ College of Plant Protection, Southwest University, Chongqing, China; ^2^ Department of Bioengineering, University of California, Berkeley, Berkeley, CA, United States; ^3^ Department of Plant & Microbial Biology, University of California, Berkeley, Berkeley, CA, United States; ^4^ State Key Laboratory of Agricultural Microbiology, Hubei Hongshan Laboratory, Hubei Key Laboratory of Plant Pathology, Huazhong Agricultural University, Wuhan, China; ^5^ Institute for Bioscience and Biotechnology Research, University of Maryland, Rockville, MD, United States; ^6^ Department of Plant Pathology, China Agricultural University, Beijing, China

**Keywords:** crop protection, crop-pathogen interactions, immune response, secondary metabolites, synthetic chemicals

Crop diseases are responsible for substantial yield losses worldwide, thereby threatening global food security. In this Research Topic, a collection of high-quality articles reported recent research progress concerning genes, proteins, secondary metabolites involved in the interactions between crop plants and their pathogens as well as utilization of new synthetic chemicals in control of crop diseases. As co-editors of this research topic, we appreciate the contributions from the authors of the papers published under this topic and highlight the three themes drawn from their research findings.

## Identification of key genes and proteins in immune response and evasion

The immune response of crop plants to pathogen attacks and the immune evasion of pathogens from their host plants have attracted more and more attention in the field of phytopathology research. Jiang et al. and Zhan et al. systematically analyzed the roles of TaSnRK and TaPAL members in wheat against notorious pathogens, including *Blumeria graminis*, *Fusarium graminearum* and *Puccinia striiformis*.

*Ralstonia solanacearum* causes devastating diseases in a variety of economically important crops. Cao et al. identified 23 differentially expressed long intergenic ncRNAs (lincRNAs) in tomato inoculated with *R. solanacearum*, and predicted 171 possible target genes of these lincRNAs. Further studies suggested that lincRNAs might be involved in hormone signaling pathways and regulation of AGO protein expression in response to *R. solanacearum*. An et al. showed that a type III effector RipTPS from the avirulent *R. solanacearum* strain specifically induced cell death in *Nicotiana tabacum* but not in *N. benthamiana*. Three amino acid residues in RipTPS have been identified to be critical for the recognition of RipTPS_G_ in *N. tabacum*.

Huanglongbing (HLB), caused by *Candidatus* Liberibacter asiaticus (*C*Las), is the most devastating citrus disease. Basu et al. discovered that two *C*Las proteins, LasP235 and Effector 3, interact with and inhibit the functions of multiple citrus proteins involved in the production of reactive oxygen species and antibacterial metabolites as well as programed cell death.

## Characterization of secondary metabolites involved in crop-pathogen interactions

Various secondary metabolites of plants are involved in the process of disease resistance. The role of the hydroxycinnamate amide (HCAA) pathway in plant immunity was reviewed by Liu et al. in three layers: biosynthesis of HCAAs from the phenylpropanoid pathway, function of HCAAs in plant disease resistance, and regulatory pathways of HCAAs accumulation. This review refines the mechanisms by which various secondary metabolites of plants are involved in immune responses and points the way to future exploration of phytochemical defense metabolites.

As one of the lipophilic secondary metabolites deposited in the plant cell wall, suberin is an essential component of the Casparian strip of the root endodermis. Chen et al.reviewed the function of plant root suberin lamella as a physical barrier in defense against biotic and abiotic stresses in four layers: establishment of suberin lamellae in the cell wall, biosynthesis of suberin, deposition of suberin in cell wall, and stress tolerance.

Secondary metabolites play important roles in plant pathogens too. Ralsolamycin, one of secondary metabolites in *R. solanacearum*, is known to be involved in crosstalk between *R. solanacearum* and fungi. Li et al. demonstrated that Ralsolamycin biosynthesis can be oppositely regulated by PhcA and PhcR in *R. solanacearum* to respond and adapt to changing environmental conditions.

## Utilization of new synthetic chemicals for crop disease control

The development and utilization of novel fungicides with novel structures, high efficiency and low toxicity are of great importance for crop disease control. Yao et al. designed and synthesized a series of 1,3,4-oxadiazole derivatives from benzoyl hydrazine and aromatic aldehydes. Compared to control fungicide carbendazim, most of the oxadiazole derivatives exhibited enhanced activities against three fungal pathogens of maize, especially *Exserohilum turcicum*. Molecular docking illustrated that oxadiazole derivatives can bind to the active site of succinate dehydrogenase (SDH) through hydrophobic contacts and hydrogen bonds, indicating that 1,3,4-oxadiazole derivatives are promising candidates for fungicide development.

As an important agrochemical, nitrogen fertilizer plays a central role in plant growth and fitness, and some specific forms of nitrogen fertilizer may also improve plant resistance. Maywald et al. found that application of nitrate or cyanamide instead of ammonium to wheat plants significantly suppressed two important wheat fungal pathogens, i.e., airborne *B. graminis* and soilborne *Gaeumannomyces graminis*. They also observed relatively higher abundance of fungal pathogenic taxa in wheat plants fertilized with ammonium. These interestingly observations suggest that nitrate and cyanamide fertilization could reduce the severity of some fungal diseases in wheat.

Additionally, biocontrol of plant diseases is an alternative disease management strategy that is environmentally benign, durable, and compatible with other control measures. Xu et al. identified a new *Bacillus amyloliquefaciens* strain XY-1 that exhibits antagonistic activity against a variety of phytopathogens. For example, antagonism tests using wheat spikelet inoculation showed that strain XY-1 displayed strong antifungal activity against *F. graminearum*, indicating that strain XY-1 can be used as a biological agent in the field to control Fusarium head blight.

In conclusion, these articles on this Research Topic have collectively given readers a snapshot of the most recent advances in research towards improving crop health. We hope that the findings presented in these articles will not only provide useful new information for specific research projects, but also help attract additional researchers to this rapidly expanding field of crop protection.

## Author contributions

All authors listed have made a substantial, direct, and intellectual contribution to the work and approved it for publication.

